# Supplementary material for: PrEP discontinuation, cycling, and risk: Understanding the dynamic nature of PrEP use among female sex workers in South Africa
Source: PLoS One. 2024 Sep 26;19(9):e0310489. doi: 10.1371/journal.pone.0310489 (PMC11426525; doi:10.1371/journal.pone.0310489)
Supplement: Attachment — Submitted filename: Reviewers comments-PONE-D-23-32704.docx [file pone.0310489.s001.docx]

**Reviewer’s comments**

This is a very interesting study that provides valuable insights into PrEP cycling among female sex workers in South Africa. The manuscript is well written but there are a few major and minor comments that need to be addressed to further improve it.

**Major**

1. The introduction is well written and the issue of PrEP cycling despite sustained high risk of HIV acquisition is clearly shown. The aim of the study is also clear although there seems to be a slight disconnect between the gap and the aim. You indicate in line 61-62 that “less is known about whether women who discontinue PrEP choose to later re-initiate and what potential motivators and key factors may influence these decisions”. The aim does not seem to be specific to the PrEP cyclers, but it would be good to have this connection between the gap and the aim by adding a word on ‘PrEP cycling’ to your aim in line 66-68 to keep focus.
2. In your methods, a) Give a little bit more detail on the implementation of the *Siyaphambili* trial and how this supplement study contributed to the *siyaphambili* trial for the reader to know how this study fitted in or how the two studies were connected (line 72-74).

b) Was there further screening/assessment of eligibility when the FSW were referred. If not, how did you minimize sample bias considering that referrals were done by the organization staff, whose services were somehow being assessed through FSW experiences? Add some bit of detail to the recruitment processes, since some participants were those who did not initiate/get one-month refills.

c) At what point did the mobile staff refer them for interviews and where would they find them (did they find and give them referrals during their mobile outreaches, or did they give phone contacts or other locator information to the study staff? Please indicate these details (line 83-84

d) Line 84-85 indicates that interviews were conducted at sex work venues or HIV TB Care’s drop-in centre. What guided you in deciding the interview venue for the different interviews.

e) Did you use the same interview guide for FSW and the Key informants? Please indicate.

f) Line 95 indicates giving incentives, which do not conform with the GCP guidelines. I would suggest that we call this a compensation (it could be for their time/transport)

g) Line 92-93 is a bit confusing, when you say isiZulu interviews were simultaneously translated to English, I get a feeling there was no isiZulu transcript but a direct audio translation from the local language to English. So, this comparison between the original isiZulu and English transcripts is a bit confusing. Please cross-check this. Also indicate who conducted the interviews.

h) You indicate in the analysis (line 99-100) that the lager team reviewed RAFs. Who constituted the lager team? Please show a brief composition of the study team. Also remember to indicate a statement on authors’ contributions.

i) In your data analysis, you mention RAFs, I presume these followed a specific thematic format since you indicated that your analysis was done deductively and inductively, and that your results followed/ were guided by Baral’s modified social ecological model (MSEM). In that respect, you need to clearly indicate at what point in your research you employed the model. Were your data tools designed in respect to the model?

j) Line 101 on data analysis, I suggest that you indicate this as ‘detailed analysis’ since you had already mentioned analysing your data rapidly, so that it does not get confusing. Line 105-109 lacks clarity of how the analysis process was done. The reviewing of coded transcripts and the summarizing of sub-themes is quite confusing. Indicate the overarching themes that you identified.

3. *Results*- I would suggest that you systematically structure your results following the MSEM like you indicated in your introduction and analysis. Starting with the text on key issues that emerged (line 132- it should actually start from line 129 as knowledge is an individual level factor), I suggest that you clearly organize these results under the different levels of the MSEM, where they each fit, followed with supporting quotes. In that case, you will not need a separate table for quotes, you could instead provide a table for KI demographics.

**Minor**

1. The predetermined criterion in the methods section line is well indicated to show the categories of FSWs that were included. You however need to show how the PrEP outreach staff identified/arrived at the participants. For example, did they scan through the different records, etc.
2. Line 229-239 in your results, you indicate stigma as a key motivator to temporarily discontinue PrEP. I would think that anything that pushes one to discontinue PrEP, would be a demotivator, barrier or challenge.
3. You need to do some formatting to the table to separate the variable subheadings from the values/categories assigned to them (e.g. if age is bolded, then remove the bold from the age values/categories).
4. Also to avoid repetitions and reduce the number of words, remove words like majority/ most (line 113, 114), since you already have the exact figures indicated there.
5. In reference to my earlier concern of referrals, how did you ensure that you interviewed the right people or categories of people if you did not reassess eligibility. I see a risk of having a biased sample or one that has been coached. I am wondering if this was a limitation.
