# Supplementary material for: PrEP discontinuation, cycling, and risk: Understanding the dynamic nature of PrEP use among female sex workers in South Africa
Source: PLoS One. 2024 Sep 26;19(9):e0310489. doi: 10.1371/journal.pone.0310489 (PMC11426525; doi:10.1371/journal.pone.0310489)
Supplement: Attachment — Submitted filename: Final review.docx [file pone.0310489.s003.docx]

Final review (reviewer #1)

All initial comments to the author(s) have been addressed to my satisfaction. Just one minor observation in the response to comment (h line 112), I do not think it is necessary to mention the name of the interviewer here, initials are enough. Otherwise, thanks for addressing the comments.
